# Modelling environmental factors correlated with podoconiosis: a geospatial study of non-filarial elephantiasis

**DOI:** 10.1186/1476-072X-13-24

**Published:** 2014-06-20

**Authors:** Yordanos B Molla, Nicola A Wardrop, Jennifer S Le Blond, Peter Baxter, Melanie J Newport, Peter M Atkinson, Gail Davey

**Affiliations:** 1 Brighton and Sussex Medical School, Falmer, Brighton BN1 9PS, UK; 2 Geography and Environment, University of Southampton, Highfield Campus, Southampton SO17 1BJ, UK; 3 Department of Earth Sciences, Natural History Museum, Cromwell Road, London SW7 5BD, UK; 4 Institute of Public Health, University of Cambridge, Cambridge CB2 2SR, UK

**Keywords:** Podoconiosis, Spatial analysis, Epidemiology, Soil, Ethiopia

## Abstract

**Introduction:**

The precise trigger of podoconiosis — endemic non-filarial elephantiasis of the lower legs — is unknown. Epidemiological and ecological studies have linked the disease with barefoot exposure to red clay soils of volcanic origin. Histopathology investigations have demonstrated that silicon, aluminium, magnesium and iron are present in the lower limb lymph node macrophages of both patients and non-patients living barefoot on these clays. We studied the spatial variation (variations across an area) in podoconiosis prevalence and the associated environmental factors with a goal to better understanding the pathogenesis of podoconiosis.

**Methods:**

Fieldwork was conducted from June 2011 to February 2013 in 12 *kebeles* (administrative units) in northern Ethiopia. Geo-located prevalence data and soil samples were collected and analysed along with secondary geological, topographic, meteorological and elevation data. Soil data were analysed for chemical composition, mineralogy and particle size, and were interpolated to provide spatially continuous information. Exploratory, spatial, univariate and multivariate regression analyses of podoconiosis prevalence were conducted in relation to primary (soil) and secondary (elevation, precipitation, and geology) covariates.

**Results:**

Podoconiosis distribution showed spatial correlation with variation in elevation and precipitation. Exploratory analysis identified that phyllosilicate minerals, particularly clay (smectite and kaolinite) and mica groups, quartz (crystalline silica), iron oxide, and zirconium were associated with podoconiosis prevalence. The final multivariate model showed that the quantities of smectite (*RR* = 2.76, 95% *CI:* 1.35, 5.73; *p* = 0.007), quartz (*RR* = 1.16, 95% *CI*: 1.06, 1.26; *p* = 0.001) and mica (*RR* = 1.09, 95% *CI:* 1.05, 1.13; *p* < 0.001) in the soil had positive associations with podoconiosis prevalence.

**Conclusions:**

More quantities of smectite, mica and quartz within the soil were associated with podoconiosis prevalence. Together with previous work indicating that these minerals may influence water absorption, potentiate infection and be toxic to human cells, the present findings suggest that these particles may play a role in the pathogenesis of podoconiosis and acute adenolymphangitis, a common cause of morbidity in podoconiosis patients.

## Background

Podoconiosis is endemic non-filarial elephantiasis of the lower legs of people that have prolonged barefoot exposure to red clay soil. Globally, an estimated four million people are affected by podoconiosis in tropical Africa, central and South America, and northwest India [1]. Previous studies have suggested various environmental triggers that may cause podoconiosis [2-5]. In particular, soil type, geological characteristics of the underlying deposits, altitude and rainfall have been correlated with disease occurrence [2,3]. Furthermore, disease prevalence was found to be higher among people who did not routinely wear shoes, suggesting causal relationships between podoconiosis, the environment, and lifestyle [2].

Several studies have evaluated the links between environmental factors, particularly soil, and podoconiosis, the findings of which have provided data hinting at the potential cause of podoconiosis [2-4,6,7]. However, these studies have several limitations. Some have been based on subjective assessments: for example, the association between “red soil” and podoconiosis was based on Price’s visual observation of comparatively higher disease prevalence within a 25 km radius of soils with a reddish colouration. The red colour of the soil was considered to be due to an increased iron oxide content based on classifications of lateritic or ‘ferrisol’ soils [2]. Generally, the sample sizes used in the published research were small: for example, Price *et al.*[5] and Price and Henderson [7] postulated an association between podoconiosis and elements within the soil, such as silicon (Si) and aluminium (Al), from observing particles containing these elements in the lymphatic tissues of 38 individuals in Ethiopia and 17 in Cameroon. Although the soils in these disease-endemic areas were analysed, the association between soil type and disease prevalence in the same areas was not studied. Additionally, studies examining soil properties in relation to podoconiosis have delivered varying results. For example, Price *et al*. [7] suggested that silicon (Si) and aluminium (Al) may play a role in disease initiation in Ethiopia since the Al/Si ratio varied between tissues of elephantiasis cases and controls. However, a later study by Price *et al*. [6] in Cameroon did not show a significant difference. Frommel *et al.*[8] identified zirconium (Zr) as an important factor. More recently, Harvey *et al.* suggested that the effects of the amorphous silicates commonly found in podoconiosis-endemic areas were not comparable with those of crystalline silica, and that amorphous silica was unlikely to cause podoconiosis [9].

These conflicting findings highlight the need for a comprehensive exploration of soil characteristics, including those that have been linked previously to the disease, to fully elucidate the potential causes of podoconiosis. Understanding these soil properties will be fundamental to solving the longstanding puzzle of the pathogenesis of podoconiosis. In the present study, we undertook an exploratory analysis to examine the relationships between observed podoconiosis prevalence and a range of potential environmental covariates (including soil characteristics, underlying geology, altitude, precipitation, slope and water flow accumulation). From this we developed a model that identified the candidate environmental factors that correlate significantly with podoconiosis.

## Results

### Secondary predictors of podoconiosis

**Figure 1:**
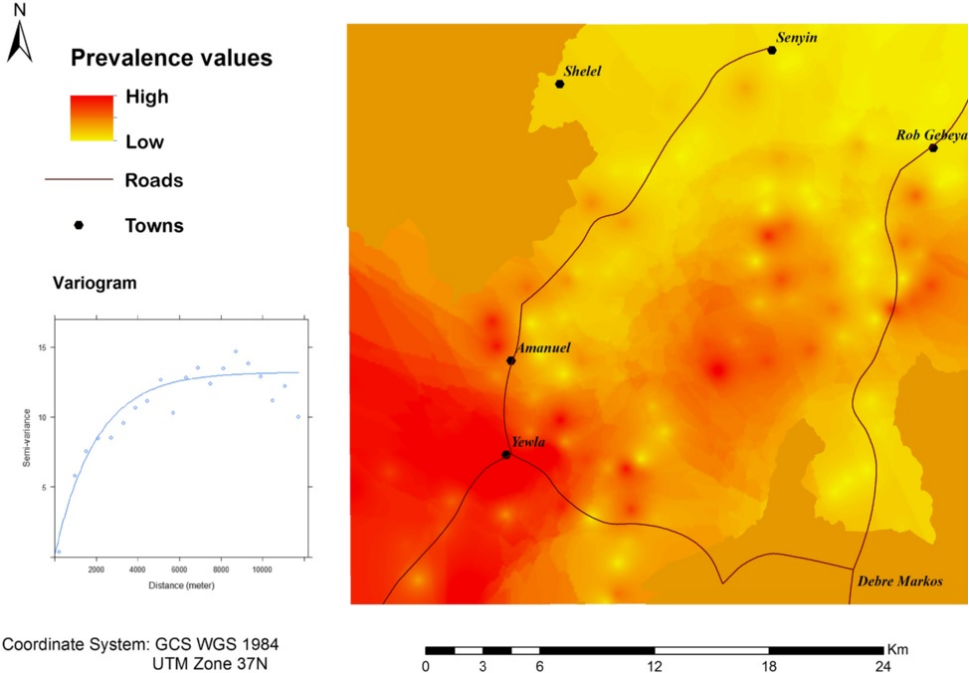
Spline interpolation of podoconiosis prevalence distribution and variogram fitted with an exponential model*. *The intensity in podoconiosis prevalence increases from yellowish to reddish.

**Figure 2:**
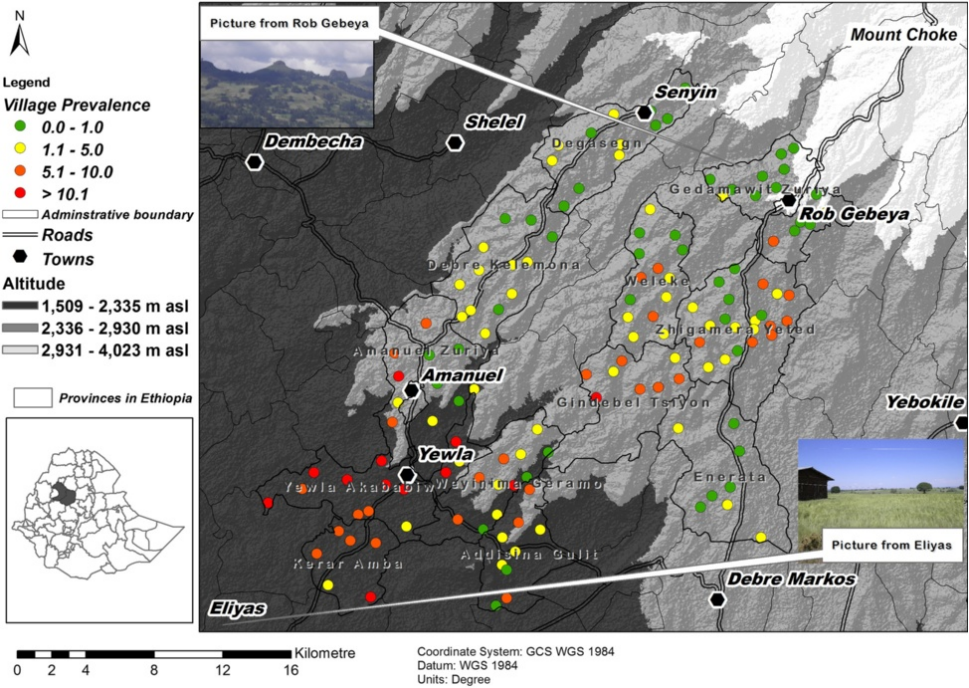
Spatial distribution of podoconiosis prevalence and elevation in East Gojam of northern Ethiopia**. **The prevalence varied from zero to greater than ten percent, and the altitude in the area ranged from 1500 m asl (above sea level) to more than 4000 m asl.

**Figure 3:**
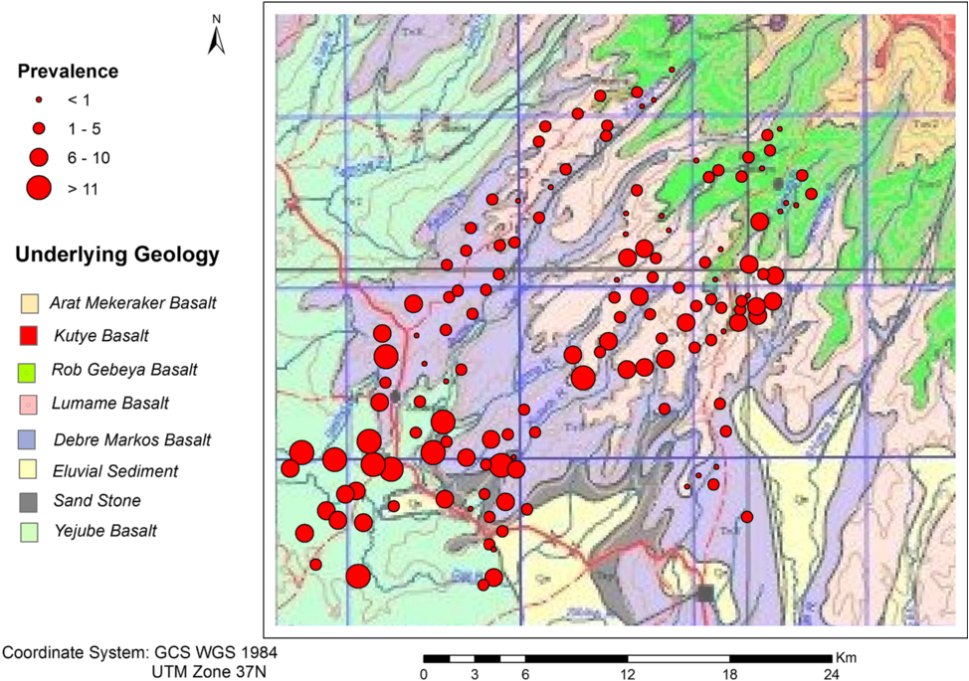
Underlying geology according to Ethiopia Geological Survey and podoconiosis prevalence (in percent) distribution sampled at 147 villages within East Gojam, northern Ethiopia.

The sample variogram of podoconiosis prevalence exhibited clear spatial autocorrelation and was fitted well by an exponential model (Figure 1). Crude exploratory spline interpolation indicated an increase in the prevalence of podoconiosis from the north-east (at the summit of Mount Choke) towards the south-west of the study area (Figure 1).The increase in podoconiosis prevalence from the north-east to the south-west (Figure 1) was matched by a decrease in altitude and precipitation in the same direction (see Figure 2 for altitude). The altitude in the study area ranged from 2000 to 4000 m above sea level (asl) and the mean annual precipitation ranged from 1000 to 1200 mm. In addition, the underlying geology of the study area included igneous deposits that were classified into eight groups (reflecting the variation in deposit characteristics) and varying values of prevalence were recorded within the same deposit type (Figure 3).

The observed variations in altitude, precipitation and geology were not, however, analysed further in direct association with podoconiosis. This is because, in addition to the indirect effect of these factors on the development of podoconiosis (which we account for by predicting soil properties), (1) the lowest altitude (2100 m asl) in the study area was higher than many places where podoconiosis has previously been recorded [10]; and (2) the smallest mean precipitation value in the study area (997 mm) was large compared with previously suggested levels of precipitation associated with podoconiosis (>1000 mm) [10]. Therefore, these variables were considered in predicting soil characteristics in a first step based on regression kriging, before exploring the relations between podoconiosis and the soil characteristics.

### Primary predictors of podoconiosis

**Figure 4:**
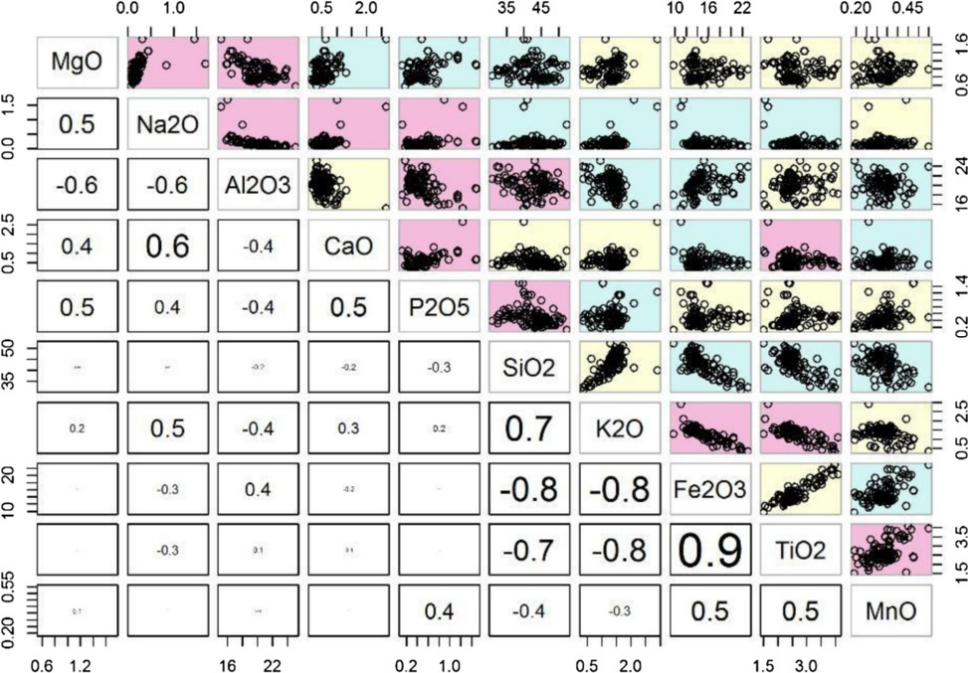
Spearman’s correlation matrix of bulk soil chemicals (major oxides).

Most of the soil characteristic variables were not normally distributed, and multiple variables were significantly correlated as depicted in Figure 4 (scatter plot and correlation coefficient above and below the diagonal respectively). The soil variables that were retained for further analysis were: iron oxide (Fe_2_O_3)_ and zirconium (Zr), proportion of particles within the soil (soil particle size <1 *μm,* analysed using water as a dispersant), quartz (crystalline silica), clay minerals (smectite, kaolinite), mica and chlorite. It should be noted that the kaolinite-smectite (K-S) phases of the soil were classified as smectite (if the K-S mixed layer was dominantly smectite rich) or kaolinite (if the K-S was dominantly kaolinite rich).

Geological deposit types in the study area (Figure 3) were not considered as covariates in the regression kriging of soil characteristics, as they did not adequately capture the spatial heterogeneity of the soil within the study area. The majority of the soil variables showed significant correlations (according to *p*-values from linear regression analysis) with altitude and precipitation. Since altitude and precipitation were highly correlated (*r* = 0.9), altitude was selected for regression kriging.

The predicted soil characteristics were extracted for the 108 villages for which disease prevalence values were available (this excluded locations where the kriging standard error was greater than the mean). Univariate and multivariate regression analysis were carried out to analyse the associations between podoconiosis prevalence and the interpolated soil characteristics for the 108 villages.

The univariate and multivariate associations between disease prevalence and soil characteristics are shown in Table 1. Multivariate analysis indicated that particle size, Fe_2_O_3_, Zr and kaolinite were not significantly associated with disease prevalence. However, particle size was retained in the multivariate model as previous evidence has suggested a role in disease causation [11]. Multi-collinearity assessment estimated that Fe_2_O_3_ had a Variance Inflation Factor (VIF; a VIF greater than 10 indicates multi-collinearity between variables) of greater than 10, so this variable was eliminated and multivariate analysis continued with the remaining soil characteristic variables, soil particle size < 1 *μm*, quartz, mica and smectite. All variables apart from soil particle size <1 *μm* were significantly associated with prevalence with a VIF of <5 in the final multivariate model (Table 1).

**Table 1:** Univariate and multivariate regression of village prevalence of podoconiosis and soil characteristics analysed in this study

**Univariate analysis**					**Multivariate analysis**			
** Soil variable**	**Estimate**	***p-*value**	***OR***	*** CI***	**Estimate**	***p-*value**	***OR***	*** CI***
**Quartz**	0.18	*p < 0.001*	1.21	(1.12,1.30)	0.14	*p* ***=*** 0.001	1.16	(1.06, 1.26)
**Mica**	0.09	*p < 0.001*	1.09	(1.07, 1.12)	0.09	*p < 0.001*	1.09	(1.05, 1.13)
**Fe**_**2**_**O**_**3**_	−0.62	*p < 0.001*	0.54	(0.46, 0.63)	NA^a^			
**Particle size < 1** ***μ*m**	0.04	*p* ***=*** 0.526	1.04	(0.92, 1.17)	−0.15	*p* ***=*** 0.058	0.86	(0.73, 1.00)
**Zirconium**	0.02	*p < 0.001*	1.02	(1.02, 1.03)	NA^b^			
**Smectite**	0.88	*p* ***=*** 0.022	2.42	(1.11, 4.94)	1.04	*p* ***=*** 0.007	2.76	(1.35, 5.73)
**Kaolinite**	−0.09	*p* ***=*** 0.002	0.91	(0.86, 0.97)	NA^b^			

**Note**: Number of villages included = 108.

CI = Confidence interval, RR = Risk Ratio.

^a^Iron oxide excluded: VIF of >10;

^b^Zirconium, and Kaolinite excluded: *p-*values greater than 0.050.

The final model included smectite (*RR* = 2.76, 95% *CI*: 1.35, 5.73; *p* = 0.007), quartz (*RR* = 1.16, 95% *CI*: 1.06, 1.26; *p =* 0.001) and mica (*RR* = 1.09, 95% *CI*: 1.05, 1.13; *p* < 0.001), each of which displayed positive correlations with podoconiosis prevalence in the study area. The model presented in *Equation* 1 represents the relationship between the logarithm of podoconiosis case count (log *ỹ*) and the three soil minerals:

(1) $$\begin{array}{ll} \log\;\tilde{y}\;= & ‒8.25\;+\;0.14\;\mathrm{quartz}\;+\;0.09\;\mathrm{mica}\\ & +\;1.04\;\mathrm{smectite} \end{array}$$

Hence, keeping all other factors constant, podoconiosis case count (*ỹ*) becomes:

(2) $$\begin{array}{ll} \;\tilde{y}\;= & 2.64*10^{‒4}\;+\;3.04*10^{‒4}\;\mathrm{quartz}\;+\;2.89\\ & *10^{‒4}\;\mathrm{mica}\;+\;7.5*10^{‒4}\;\mathrm{smectite} \end{array}$$

Podoconiosis count alone = *exp*^*-8.24*^ = 2.64*10^*−4*^

Podoconiosis count considering increase in quartz = *exp*^*-8.24+ 0.14*^ = 3.04*10^*−4*^

Podoconiosis count considering increase in mica = *exp*^*-8.24+ 0.09*^ = 2.89*10^*−4*^

Podoconiosis count considering increase in smectite = *exp*^*-8.24+ 1.04*^ = 7.5 *10^*−4*^

Based on the model, when the percentage of smectite in the soil increased by 1% (e.g., a rise from 1% to 2% in the value across its full range), the podoconiosis case count almost trebled (*Equation* 2). Similarly, the equivalent increase in the podoconiosis case count was 9.47% for mica and 15.15% for quartz. This assumption, based on the calculations, held true when all other factors contributing to the development of podoconiosis (such as genetic susceptibility, an individual’s behaviour in terms of foot-washing and shoe-wearing practices) were held constant.

**Figure 5:**
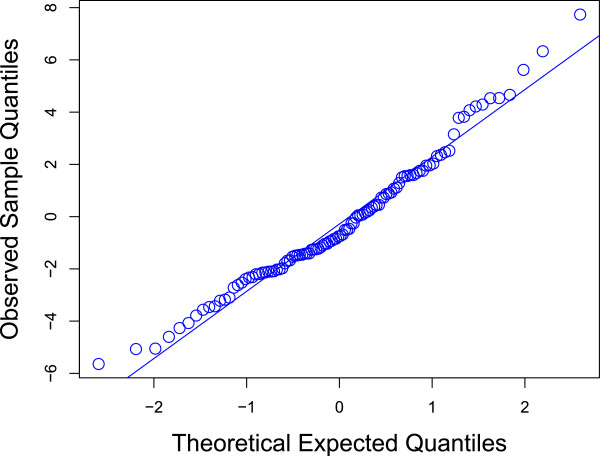
Normal Q-Q plot for the observed sample against theoretical quantiles.

The coefficient of determination for the model was *r*^2^ = 0.4 which indicated that the covariates in the final multivariate regression model accounted for approximately 40% of the variation in the outcome variable. The model’s goodness-of-fit was tested by examining the residual deviance and comparing the deviance with a Chi-square distribution. The residual deviance test of the model gave a value of 6.8, while comparison of the residuals with a Chi-squared distribution gave zero (8.86 × 10^–89^). The Quantile-Quantile (Q-Q) plot (Figure 5) confirmed that the empirical data were sufficiently close to the theoretical reference line, indicating a reasonably good model fit.

## Discussion

This study identified specific soil characteristics associated with increased prevalence of podoconiosis. Using environmental and individual data across an area with a wide spectrum of podoconiosis prevalence, the study filled the research gap from previous studies where the soil composition was not directly assessed in areas where study participants lived [5], or where soil composition was assessed in areas dichotomized into endemic or non-endemic, based on expert opinion and without prevalence data [7]. In addition, recent developments in soil chemical analysis techniques and advances in geospatial and statistical methods enabled us to explore environmental data more extensively than was possible previously.

We found that the prevalence of podoconiosis was positively associated with the quantities of smectite, mica and quartz (crystalline silica) measured in the soil. The correlation between podoconiosis and smectite was larger than that between podoconiosis and other phyllosilicate minerals within the soil (kaolinite, chlorite and mica) or quartz. Smectite is classified as a 2:1 phyllosilicate, with a structure in which two tetrahedral silicate sheets sandwich a central octahedral silicate sheet. In general, 2:1 phyllosilicates have large surface areas, are able to undergo isomorphous substitution (ion exchange within its structure) and hence have elevated surface reactivity. The presence of 2:1 phyllosilicates in soils typically results in the deposits displaying distinctive characteristics such as high cation exchange capacity and shrink-swell properties. Smectite has unique properties of water absorption and expansion, and is able to modify water flow [12].

The biological properties of smectite have mostly been investigated in relation to gastrointestinal and dermatological therapeutic effects. Smectites are known as dermatological protectors because of their ability to adhere to the skin, form a protective film and absorb greases and toxins [13,14]. However, these properties of adherence and water absorption might potentially, through establishment of an external water gradient influencing permeability of the stratum corneum, increase transdermal uptake of potential toxins [15]. Release and transdermal uptake of a range of ions bound to pelotherapy clays has been demonstrated [16], so it is possible that ionic species adsorbed to clays of podoconiosis endemic areas are exchanged across the skin of the lower leg and foot. A study of the role of inflammatory biomarkers for development of podoconiosis has indicated increased anion (O_2_^−^) and hydroxyl radicals (HO) in the early stages [17]. The major source of these peroxides was suggested to be activation and subsequent elimination of macrophages, which might relate to soil minerals with high oxidation properties.

Other studies have investigated the effects of clays on infection. Montmorillonite, a form of phyllosilicate within the smectite group that typically results from the weathering of volcanic ash, was noted in the 1970s to have a greater infection potentiation effect than other phyllosilicates (kaolinite and illite). The mechanism was later explained to be the direct cytotoxic effect of montmorillonite on neutrophils, weakening cell immunity and facilitating bacterial proliferation [18,19]. A very recent study, in 2013, suggests that montmorillonite facilitates the survival of strains of enteropathogenic bacteria (microorganisms causing diseases of the intestine) in the soil by providing mineral nutrients and enabling respiratory simulation [20]. On the other hand, other studies have demonstrated the bactericidal effects of hydrated clay in which cell death occurs by “exchange of soluble clay constituents toxic to the bacteria” [21,22]. Williams *et al.*[22] noted that expandable clay minerals, particularly illite-smectite, had the most pronounced antibacterial properties due to extreme pH and Fe concentration, and Otto and Haydel [21] demonstrated that illite-smectite rich clay mixtures acquire powerful antibacterial activity due to their positive correlation with concentration of Cu^2+^ and Zn^2+^ ions, rather than their negative correlation with Fe^3+^ or lack of correlation with pH. These effects of clay mineral types in infection and our finding of strong associations between podoconiosis and soil smectite concentrations may help explain the pathogenesis of episodes of super-infection and acute adenolymphangitis (a frequent complication of podoconiosis characterized by hot, painful, and reddened swelling of the lymphedematous legs) [23-25].

Mica and quartz were also significantly associated with increased prevalence of podoconiosis. Previously, several researchers had independently observed the presence of clay minerals such as kaolinite and smectite in the soil samples they analysed [4,5,7,8]. Price *et al.* found amorphous silica and aluminium oxides in the lymph nodes of podoconiosis cases and postulated that these minerals, particularly silica, may be involved in the pathogenesis of podoconiosis [4,5]. Frommel *et al.* suggested that the high level of trace elements, such as Zr, within the soil found in podoconiosis-endemic areas was responsible for the development of podoconiosis [6-8]. Several studies lend biological plausibility to our finding of positive associations between quartz (a form of crystalline silica ubiquitous in the environment) and prevalence of podoconiosis: quartz (i) has been demonstrated to induce an inflammatory response and fibrosis in the pathogenesis of lung silicosis [26-28]; (ii) is listed as a Group 1 carcinogen by the International Agency for Research on Cancer [29]; (iii) has been shown to be more toxic to the human body than amorphous silica [9]; and (iv) animal models have shown lymphatic fibrosis and blockage comparable to that found in podoconiosis on injection of crystalline silica suspension into the lower limbs of rabbits [30].

Multiple studies indicate that zirconium is unlikely to have a pathologic role in the human body [31,32]. Our univariate analysis finding of elevated zirconium in podoconiosis-endemic areas may suggest that this element plays another role, such as in facilitating dryness and cracking of skin on the feet. A recent study in Ethiopia showed that podoconiosis cases had lower stratum corneum hydration than unaffected controls, resulting in skin dryness and cracking which, in turn, may facilitate the ingress of mineral particles or microorganisms through the skin barrier [23]. The ability of zirconium to accumulate in skin stratum corneum and sometimes to cause skin granuloma (inflammation) has been documented [33,34]. Adding zirconium to commonly used aluminium chloride antiperspirants increases antiperspirant efficacy [35], and application of non-emollient antiperspirants was also shown to reduce sweat moisture in feet [36]. Zirconium is a trace element in soil and so exists in very small amounts, thus our observation may simply reflect the association of zirconium with other soil elements. However, it is also possible that chronic exposure to zirconium in the soil plays a role in dehydration and cracking of the skin of the feet, thereby predisposing to podoconiosis.

Our model captured 40% of the variation in podoconiosis prevalence. The regression residuals indicate that the development of podoconiosis depends on etiological factors other than purely environmental factors. Genetic susceptibility has been shown to be another key aetiological factor [37], as have shoe-wearing practices [37,38]. This study had limited capability to capture individual behaviours, disease severity and genetic variations in relation with environmental factors that are also likely to have been important factors in explaining the residual variation [39,40]. However, the soil composition factors identified by our analyses will be valuable to enhance our understanding of the development of podoconiosis in areas where the majority of people walk barefoot.

## Conclusion

The findings of this study challenge some of the pre-existing assumptions that link podoconiosis occurrence with the presence of compounds and elements within the soil [6-8]. Our study narrows the focus to phyllosilicate clay minerals, in particular smectite, and quartz (crystalline silica). Our findings contribute to the growing research on the aetiology of podoconiosis, and provide new starting points for further exploration of podoconiosis aetiology using biomedical and toxicological studies.

## Methods

### Study area

The study was conducted in Gozamen and Machakel districts of East Gojam Zone (province) in northern Ethiopia. Twelve *kebeles* (representing the lowest administrative units) with 147 villages, distributed over an area of 900 km^2^, were covered in the study (Figure 2) from June 2011 to February 2013.

### Data sources and preparation

#### Case data

Podoconiosis case data were collected by Health Extension Workers (HEW; community health workers in rural Ethiopia) who were trained to identify people displaying the symptoms of podoconiosis in their catchment *kebeles*. Following training, the HEWs visited each household and recorded the following: number of members residing within the household, number of persons with podoconiosis (cases), and sex and age of all household members using a standardised checklist [25]. Based on the presence or absence of podoconiosis cases in a house, the overall household was either classified as a case or control household. Village level prevalence was calculated based on the number of adult (>15 years old) cases in all households among the adult population within the same village. Data were entered into a spread-sheet and imported into the R statistical program (version 2.15).

#### Soil data

Soil samples were taken from villages selected randomly from six of the twelve *kebeles*. An initial control or case household was selected randomly and then every third household in the village was included for soil sampling. Soil samples were collected from selected households, and from traverses connecting main *kebeles* and towns in the study area. At each sample site, the GPS (Geographic Positioning System) coordinates and altitude at the centre of the field were recorded and sample bags were labelled with the identification number given to the household. Additional soil samples were collected from traverses and frequently used paths, with sampling sites selected for every 100 m vertical descent or 1 km road distance from the point of maximum altitude (Mount Choke). In total, soil samples from 86 sample sites, within 31 villages, were included in the analysis for a range of geochemical and mineralogical characteristics, using instruments based at the Natural History Museum, London (For details refer to Additional file 1, Table 1 and (Le Blond et al: Weathering of the Ethiopian plateau flood basalt, NW Ethiopia: A new weathering index to characterize and compare soils, submitted [41]).

#### Spatial and additional environmental data

Podoconiosis prevalence and soil characteristic data were geo-referenced based on GPS coordinates recorded in the field during soil sampling, and coordinate data obtained from the Ethiopian Mapping Agency (EMA). Detailed topographic information and a geological map of the study area (with 1:50,000 and 1:250,000 scale, respectively) were also obtained from the EMA and Ethiopian Geological Survey. Precipitation and temperature data, with a spatial resolution of 1 km, compiled monthly over a period of 50 years (from 1950–2000), were extracted to the village level in ArcGIS from an online meteorological source (http://www.worldclim.org). In addition, digital elevation model (DEM) data were downloaded with a spatial resolution of 30 m from the NASA (National Aeronautics and Space Administration) Global ASTER (Advanced Space-borne Thermal Emission and Reflection Radiometer data repository (http://asterweb.jpl.nasa.gov/gdem.asp). Percentage slope was calculated using the DEM in ArcGIS. Similarly, the DEM was used to calculate the water flow direction and water flow accumulation. Water flow direction and accumulation take into account slope steepness and direction, both of which influence soil deposition.

### Data analysis

#### Analysis and interpolation of soil characteristics

The primary variable of interest was podoconiosis prevalence. The prevalence data were explored for the presence of a spatial pattern in the 147 villages. For this purpose, a variogram (which characterises the spatial dependence or spatial correlation between pairs of points in space) was estimated and fitted with an exponential model in the geoR package in the R statistical software. Spline interpolation was used to characterise the spatial distribution of prevalence in the study area. Spline interpolation takes as input a set of point data values and produces a smooth, spatially continuous surface which passes through the sampled data points in the area of interest [42].

Geology, altitude, precipitation and temperature influence soil formation and their influence on disease development is not direct. Therefore, these covariates were classified as secondary predictors in this study. The soil characteristics, determined from geochemical analysis, were considered as primary environmental predictors because podoconiosis arises from direct contact with the soil. Soil characteristics measured were: organic and inorganic content; major oxides; trace elements; and minerals including clay, crystalline and amorphous content.

**Figure 6:**
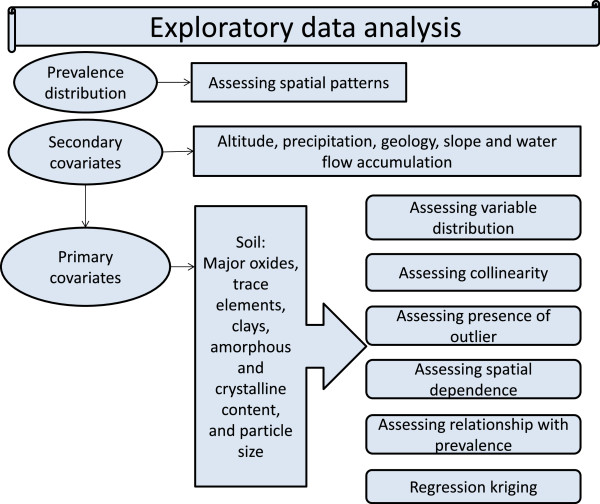
Exploratory data analysis flowchart.

**Figure 7:**
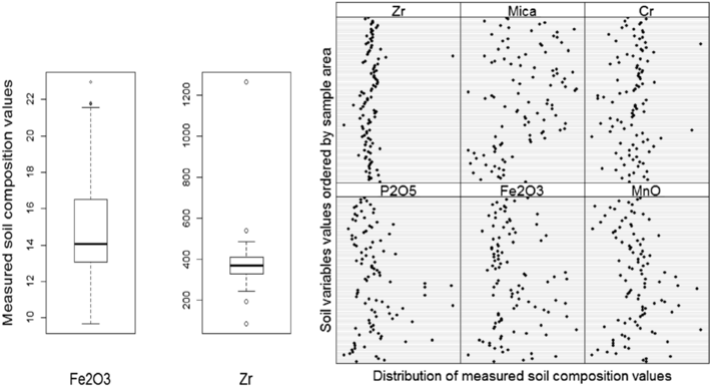
Checking for outliers using boxplot and Cleveland plots***. *** Measurement of Fe_2_O_3_ included outlier values in a box plot (left). However the Cleveland plot (right) showed values were similar to the majority, therefore all the values for Fe_2_O_3_ were retained. On the other hand, variables such as Zr and Cr included a measurement value widely dispersed from the majority and were removed from further analysis.

Exploratory data analysis was undertaken to determine which of the soil variables, or combination of variables, should be taken forward for interpolation via regression kriging and subsequent analysis as covariates of podoconiosis prevalence (Figure 6 summarizes the exploratory analysis steps explained below). As an initial step, the assumption of normality was tested for each soil variable using histogram plots, mean, median, standard deviation, skewness and kurtosis. During the second step, pairwise correlation coefficients were calculated to assess the relationships between individual soil characteristics. For variables with non-normal distributions, the non-parametric Spearman’s Rank correlation coefficient was used. Where the correlation coefficient (*r*) between two soil characteristics was >0.6 the variables were classified as correlated. Based on the correlation analysis, in tandem with careful consideration of geological understanding, several soil characteristics were omitted from further consideration. This procedure ensured that (1) collinearity within the set of variables was reduced and (2) the soil characteristics which were carried forward for further analysis were geologically relevant. The third step focused on identifying and managing outliers. Statistical outliers were identified using box plots and Cleveland dot plots. Soil variables which contained statistical outliers were transformed for enhanced visualization and outliers were removed from the data (Figure 7). During the fourth step, spatial patterns in each of the remaining covariates were assessed using a plot to show soil measurement values. Variables that exhibited a distribution unrelated to the disease were removed (Additional file 1, Figure 1). Finally, univariate regression analysis with podoconiosis prevalence as an outcome variable excluded the remaining variables with 95% confidence interval *p* >0.500 and AIC (Akaike Information Criterion – a penalised measure of the goodness-of-fit of a model) >85.5, which was the median AIC value (Additional file 1, Table 3).

The techniques involved in soil characterisation are highly labour intensive, and so fewer soil samples were analysed (86 data points within 31 villages) than the number of villages for which prevalence data were available (147 villages). In addition, due to the varied geology of the region, soil characteristics were assumed to be inherently heterogeneous with significant variation across small areas. Collecting multiple soil samples within each village was anticipated to capture this variation. This produced multiple soil measurements recorded for the same disease prevalence value of a village.

Regression kriging was used to interpolate each soil variable as a combined function of (i) regression with a set of related covariates and (ii) spatial interpolation of the residuals of the regression through kriging [43]. Kriging is a spatial interpolation technique that predicts the value of the variable of interest at an unobserved location as a weighted average of values in neighbouring locations [42]. Covariates assessed for regression kriging were geology, slope, altitude, precipitation, and water flow accumulation. The geology of the area was selected because the underlying geology represents the parent material from which soil is formed over time. Altitude, slope, precipitation and water flow accumulation were included due to their influence on weathering and soil deposition rates). The covariates used for regression kriging of each of the soil characteristics were selected using linear regression models and correlation coefficients.

Residual variograms (from the residuals of the best fitting linear regression model) were estimated and variogram models fitted for each soil characteristic variable, prior to the application of regression kriging. From the kriging results, predictions with a kriging error smaller than a predefined threshold (taken as the mean of the kriging standard error) were retained for each soil characteristic variable and those with an error larger than the threshold were omitted from further analysis. This ensured that further analysis used only soil characteristic values which were predicted with a high degree of accuracy. The output raster variables were the predicted values of each soil characteristic variable, based on geo-statistical interpolation of the soil sample data, which used covariate information to increase the accuracy of the prediction. The predicted soil variables were overlaid onto the village level disease prevalence data points, and then interpolated soil values were extracted for each village.

#### Analysis of podoconiosis prevalence based on soil characteristics

The relationship between the interpolated soil variables and podoconiosis prevalence was assessed using univariate logistic regression analysis. Variables that did not exhibit a statistically significant association with the outcome (*p* >0.05) were omitted from further analysis. Since the prevalence data were over-dispersed, a generalized linear model (GLM) of the Quasi-Poisson family was used for the multivariate analysis. Multivariate regression analysis was applied to the remaining variables and the outputs checked for multi-collinearity using the VIF. Soil variables with a VIF greater than 10 were removed prior to the multivariate analysis and the process was repeated until the remaining soil variables had a VIF less than 10.

The final multivariate regression model was evaluated for goodness-of-fit, coefficient of determination (*r*^*2*^) and residual diagnostics. The goodness-of-fit was tested by examining the residual deviance and by comparing the residual deviance to a Chi-squared (*X*^*2*^) distribution. For an acceptable model fit, the ratio of residual deviance to degrees of freedom of the residuals is expected to be close to 1. Comparing the distribution of the residuals with a Chi-squared distribution should give a value close to zero. Residual diagnostics were also checked using plots of the residual and predicted values, and Q-Q plots of the fitted and empirical value distributions. Finally, the residuals were evaluated for autocorrelation using residual variograms and spatial plots.

## Competing interests

This study was funded by the Wellcome Trust, UK, (University Award 091956). The funders had no role in the design of the study, data collection, decision to publish, or preparation of the manuscript. The authors declare no financial or non-financial competing interests.

## Authors’ contributions

YBM, MJN and GD designed the study. YBM, JLB, PB and GD did the fieldwork. JLB analyzed the soil samples; and YBM, NAW, PMA analyzed the statistical and spatial data. YBM drafted the manuscript. All authors revised the paper for substantial intellectual content. All authors read and approved the final manuscript.

## Supplementary Material

Additional file 1 Modelling of environmental factors correlated with podoconiosis in Ethiopia. Click here for file
