# Supplementary material for: Modelling environmental factors correlated with podoconiosis: a geospatial study of non-filarial elephantiasis
Source: Int J Health Geogr. 2014 Jun 20;13:24. doi: 10.1186/1476-072X-13-24 (PMC4082615; doi:10.1186/1476-072X-13-24)
Supplement: Additional file 1 — Modelling of environmental factors correlated with podoconiosis in Ethiopia. [file 1476-072X-13-24-S1.docx]

**Additional File: Modelling of environmental factors correlated with podoconiosis in Ethiopia**

Yordanos B. Molla, Nicola Wardrop, Jennifer S. Le Blond, Peter Baxter, Melanie J. Newport, Peter M. Atkinson, Gail Davey

**Table of contents**

[Additional File, Table 1: Description of the soil characteristics analysis 2](#_Toc384220308)

[Additional File, Tables 2: Spearman’s correlation for soil covariates 3](#_Toc384220309)

[Soil minerals 3](#_Toc384220310)

[Soil particle size in *µ*m - analysis done with water 3](#_Toc384220311)

[Soil particle size in *µ*m - analysis done with Na pyrophosphate de-flocculant 4](#_Toc384220312)

[Soil content of carbon, hydrogen and nitrogen 4](#_Toc384220313)

[Soil trace elements 5](#_Toc384220314)

[Additional File, Figure 1: Spatial dependence plot examples 6](#_Toc384220315)

[Additional File, Table 3: Univariate analysis for soil characteristics and podoconiosis prevalence 7](#_Toc384220316)

# Additional File, Table 1: Description of the soil characteristics analysis

| **Soil characteristic** | **Method used** |
| --- | --- |
| Organic/inorganic carbon (C), hydrogen (H) and nitrogen (N) | CHN analyser |
| Major oxides (Al_2_O_3_, CaO, Fe_2_O_3_, K_2_O, MgO, MnO, Na_2_O, P_2_O_5_, SiO_2_ and TiO_2_) and trace elements (As, Ba, Cd, Co, Cr, Cu, Ni, Pb, Sb, Sc, Sr, Y, V and Zr) | Digestion and inductively coupled plasma - atomic emission spectroscopy/ mass spectrometry  Soil chemical content measured as weight percent plus loss on ignition. Chemical composition and elements will together add up to 100%. |
| Soil minerals: Crystalline (quartz) and amorphous components (volcanic glass), and phyllosilicates (smectite, kaolinite, mica and chlorite) | X-ray diffraction: fixed and scanning geometry  Random powders, oriented mounts and glycolated samples were analysed |
| Particle size, cumulative volume of the following cut-off sizes: 1 µm (in aerodynamic diameter), 2 µm, 5 µm, 10 µm, 20 µm, 100 µm, 500 µm, 1000 µm and 2000 µm. | Laser diffraction, using both water and de-flocculant (Na pyrophosphate) as a dispersant |

# Additional File, Tables 2: Spearman’s correlation for soil covariates

## Soil minerals

|  | **Fe oxide** | **Quartz** | **Volcanic glass** | **Feldspars** | **Kaolinite** | **Smectite** | **Mica** | **Chlorite** |
| --- | --- | --- | --- | --- | --- | --- | --- | --- |
| **Fe oxide** | 1.00 | -0.490 | 0.420 | -0.223 | 0.329 | 0.396 | -0.231 | -0.159 |
| **Quartz** | -0.490 | 1.00 | -0.540 | 0.035 | -0.028 | -0.392 | -0.276 | 0.035 |
| **Volcanic glass** | 0.420 | -0.540 | 1.00 | -0.106 | -0.249 | 0.163 | 0.260 | -0.016 |
| **Feldspars** | -0.223 | 0.035 | -0.106 | 1.00 | -0.363 | -0.269 | -0.231 | 0.147 |
| **Kaolinite** | 0.329 | -0.028 | -0.249 | -0.363 | 1.00 | 0.391 | -0.605 | -0.318 |
| **Smectite** | 0.396 | -0.392 | 0.163 | -0.269 | 0.391 | 1.00 | -0.135 | -0.320 |
| **Mica** | -0.231 | -0.276 | 0.260 | -0.231 | -0.605 | -0.135 | 1.00 | -0.008 |
| **Chlorite** | -0.159 | 0.035 | -0.016 | 0.147 | -0.318 | -0.320 | -0.008 | 1.00 |

## Soil particle size in *µ*m - analysis done with water

|  | **< 1** | **< 2** | **< 5** | **< 10** | **< 20** | **< 100** |
| --- | --- | --- | --- | --- | --- | --- |
| **< 1** | 1.00 | 0.995 | 0.756 | 0.619 | 0.942 | 0.184 |
| **< 2** | 0.995 | 1.00 | 0.779 | 0.645 | 0.946 | 0.191 |
| **< 5** | 00.756 | 0.779 | 1.00 | 0.956 | 0.714 | 0.495 |
| **< 10** | 0.619 | 0.645 | 0.956 | 1.00 | 0.624 | 0.554 |
| **< 20** | 0.942 | 0.946 | 0.714 | 0.624 | 1.00 | 0.235 |
| **< 100** | 0.184 | 0.191 | 0.495 | 0.554 | 0.235 | 1.00 |

## Soil particle size in *µ*m - analysis done with Na pyrophosphate de-flocculant

|  | **< 1** | **< 2** | **< 5** | **< 10** | **< 20** | **< 100** |
| --- | --- | --- | --- | --- | --- | --- |
| **< 1** | 1.00 | 0.900 | 0.268 | 0.595 | 0.485 | 0.282 |
| **< 2** | 0.900 | 1.00 | 0.434 | 0.808 | 0.698 | 0.487 |
| **< 5** | 0.268 | 0.434 | 1.00 | 0.554 | 0.513 | 0.373 |
| **< 10** | 0.595 | 0.808 | 0.554 | 1.00 | 0.975 | 0.807 |
| **< 20** | 0.485 | 0.698 | 0.513 | 0.975 | 1.00 | 0.885 |
| **< 100** | 0.282 | 0.487 | 0.373 | 0.807 | 0.885 | 1.00 |

## Soil content of carbon, hydrogen and nitrogen

|  | **Organic carbon** | **Inorganic carbon** | **Nitrogen** | **Hydrogen** |
| --- | --- | --- | --- | --- |
| **Organic carbon** | 1.00 | 0.120 | 0.399 | 0.285 |
| **Inorganic carbon** | 0.120 | 1.00 | 0.242 | 0.453 |
| **Nitrogen** | 0.399 | 0.242 | 1.00 | 0.181 |
| **Hydrogen** | 0.285 | 0.453 | 0.181 | 1.00 |

## Soil trace elements

|  | **As** | **Ba** | **Cd** | **Co** | **Cr** | **Cu** | **Ni** | **Pb** | **Sb** | **Sc** | **Sr** | **Y** | **V** | **Zr** |
| --- | --- | --- | --- | --- | --- | --- | --- | --- | --- | --- | --- | --- | --- | --- |
| **As** | 1.00 | 0.06 | -0.10 | -0.02 | -0.19 | -0.13 | -0.20 | 0.08 | 0.15 | -0.07 | -0.03 | -0.04 | 0.13 | -0.13 |
| **Ba** | 0.06 | 1.00 | -0.46 | -0.10 | -0.25 | -0.17 | -0.29 | 0.09 | 0.02 | 0.23 | 0.64 | -0.40 | -0.01 | -0.61 |
| **Cd** | -0.10 | -0.46 | 1.00 | 0.03 | 0.47 | 0.34 | 0.15 | 0.01 | -0.02 | -0.34 | -0.29 | 0.20 | -0.26 | 0.35 |
| **Co** | -0.02 | -0.10 | 0.03 | 1.00 | -0.03 | 0.02 | -0.02 | 0.04 | 0.24 | 0.10 | -0.10 | 0.11 | -0.07 | 0.10 |
| **Cr** | -0.19 | -0.25 | 0.47 | -0.03 | 1.00 | 0.58 | 0.60 | -0.07 | -0.03 | -0.33 | 0.08 | 0.11 | -0.01 | 0.10 |
| **Cu** | -0.13 | -0.17 | 0.34 | 0.02 | 0.58 | 1.00 | 0.31 | 0.13 | 0.02 | -0.13 | 0.09 | 0.19 | 0.12 | 0.13 |
| **Ni** | -0.20 | -0.29 | 0.15 | -0.02 | 0.60 | 0.31 | 1.00 | -0.15 | -0.15 | -0.06 | -0.23 | 0.19 | 0.12 | 0.29 |
| **Pb** | 0.08 | 0.09 | 0.01 | 0.04 | -0.07 | 0.13 | -0.15 | 1.00 | 0.02 | 0.02 | -0.04 | -0.03 | -0.02 | -0.04 |
| **Sb** | 0.15 | 0.02 | -0.02 | 0.24 | -0.03 | 0.02 | -0.15 | 0.02 | 1.00 | 0.05 | 0.22 | 0.12 | 0.05 | 0.01 |
| **Sc** | -0.07 | 0.23 | -0.34 | 0.10 | -0.33 | -0.13 | -0.06 | 0.02 | 0.05 | 1.00 | 0.09 | -0.24 | 0.01 | -0.19 |
| **Sr** | -0.03 | 0.64 | -0.29 | -0.10 | 0.08 | 0.09 | -0.23 | -0.04 | 0.22 | 0.09 | 1.00 | -0.16 | -0.08 | -0.38 |
| **Y** | -0.04 | -0.40 | 0.20 | 0.11 | 0.11 | 0.19 | 0.19 | -0.03 | 0.12 | -0.24 | -0.16 | 1.00 | 0.19 | 0.68 |
| **V** | 0.13 | -0.01 | -0.26 | -0.07 | -0.01 | 0.12 | 0.12 | -0.02 | 0.05 | 0.01 | -0.08 | 0.19 | 1.00 | 0.04 |
| **Zr** | -0.13 | -0.61 | 0.35 | 0.10 | 0.10 | 0.13 | 0.29 | -0.04 | 0.01 | -0.19 | -0.38 | 0.68 | 0.04 | 1.00 |

# Additional File, Figure 1: Spatial dependence plot examples

The MnO and Sc measurement distribution exhibited higher values to the north-eastern part due to the volcanic ash around the summit of the mount Choke indicating spatial distribution unrelated with podoconiosis prevalence.


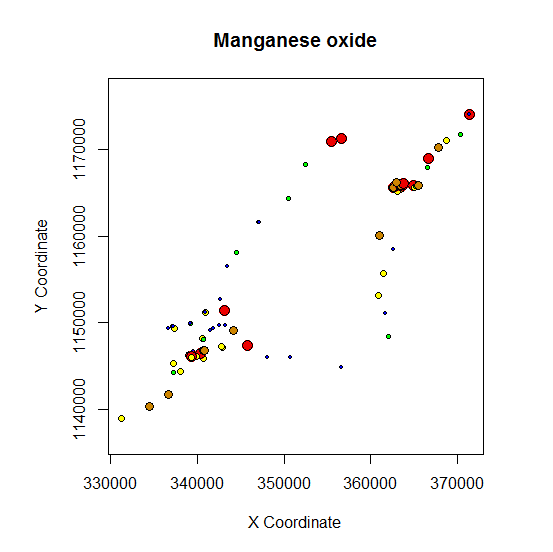

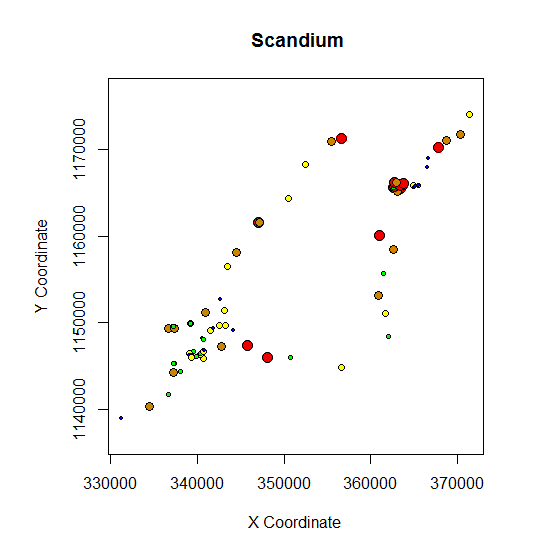


# Additional File, Table 3: Univariate analysis for soil characteristics and podoconiosis prevalence

| **Soil Characteristics** | **Univariate analysis** | | | | |
| --- | --- | --- | --- | --- | --- |
|  | **AIC** | **Estimate** | ***p* – value** | **OR** | **CI** |
| **Al2O3^Ϯ^** | 86.45 | 0.11 | 0.520 | 1.12 | (0.85, 1.49) |
| **Fe2O3** | 65.02 | -0.73 | 0.001 | 0.48 | (0.29, 0.70) |
| **Particle size <1microns- analysis by water** | 83.11 | -0.27 | 0.055 | 0.76 | (0.57, 0.99) |
| **Particle size <2microns- analysis by de-flocculant ^a^** | 87.05 | 0.01 | 0.800 | 1.01 | (0.95, 1.07) |
| **Particle size <10microns-analysis by de-flocculant ^a^** | 86.74 | 0.01 | 0.545 | 1.01 | (0.97, 1.05) |
| **Organic carbon ^Ϯ^** | 87.01 | 0.08 | 0.745 | 1.09 | (0.65, 1.83) |
| **Quartz** | 85.42 | 0.04 | 0.212 | 1.05 | (0.98, 1.13) |
| **Smectite** | 85.37 | 0.32 | 0.200 | 1.38 | (0.86, 2.34) |
| **Kaolinite** | 85.11 | -0.05 | 0.168 | 0.95 | (0.88, 1.02) |
| **Mica** | 75.28 | 0.11 | 0.003 | 1.12 | (1.05, 1.21) |
| **Chlorite** | 57.82 | -1.14 | 0.0003 | 0.32 | (0.15, 0.55) |
| **As ^Ϯ^** | 87.11 | 0.001 | 0.970 | 1.00 | (0.94, 1.07) |
| **Pb ^Ϯ^** | 87.10 | -0.007 | 0.931 | 0.99 | (0.84, 1.17) |
| **Sb ^Ϯ^** | 87.11 | -0.002 | 0.979 | 1.00 | (0.85, 1.19) |
| **V ^Ϯ^** | 87.08 | 0.001 | 0.851 | 1.01 | (0.99, 1.03) |
| **Cd ^Ϯ^** | 86.10 | 0.66 | 0.354 | 1.00 | (0.99, 1.01) |
| **Ba** | 52.41 | -0.04 | 0.0003 | 0.96 | (0.94, 0.98) |
| **Zr** | 64.50 | 0.03 | 0.0003 | 1.03 | (1.01, 1.05) |
| **Cr** | 77.23 | 0.02 | 0.012 | 1.02 | (1.01, 1.04) |
| **^a^** variables removed; AIC – Akaki Information Criterion, OR – Odds ratio, CI – Confidence interval  Variables were retained based on *p* – value < 0.500 which included AIC < 85.50 (the median AIC). | | | | | |
